# Variation in financial protection and its association with health expenditure indicators: an analysis of low- and middle-income countries

**DOI:** 10.1093/pubmed/fdab021

**Published:** 2021-04-23

**Authors:** Seun S Anjorin, Abimbola A Ayorinde, Mustapha S Abba, Oyinlola O Oyebode, Olalekan A Uthman

**Affiliations:** Population Evidence and Technologies, Division of Health Sciences, Warwick Medical School, University of Warwick, Coventry CV4 7AL, UK; Warwick-Centre for Applied Health Research and Delivery (WCAHRD), Warwick Medical School, University of Warwick, Coventry CV4 7AL, UK; Population Evidence and Technologies, Division of Health Sciences, Warwick Medical School, University of Warwick, Coventry CV4 7AL, UK; Warwick-Centre for Applied Health Research and Delivery (WCAHRD), Warwick Medical School, University of Warwick, Coventry CV4 7AL, UK; Warwick-Centre for Applied Health Research and Delivery (WCAHRD), Warwick Medical School, University of Warwick, Coventry CV4 7AL, UK

**Keywords:** catastrophic health spending, financial protection, low- and middle-income countries, out of pocket payment

## Abstract

**Background:**

An insight into variation in financial protection among countries and the underpinning factors associated with the variations observed will help to inform public health policy and practice.

**Method:**

Secondary datasets from Global Health Expenditure Database and World Bank Development Indicators collected between 2000 and 2016 were used. Financial protection was measured in 75 low- and middle-income countries (LMICs) using the sustainable development goals framework. Funnel plot charts were used to explore the variation, and regression models were used to measure associations.

**Result:**

Fifty-three (67%) countries were within the 99% control limits indicating common-cause variation; 11 countries were above the upper control limit and 15 countries were below the lower control limit. In the fully adjusted model, country, spending on health relative to their economy had the strongest association with the variation in catastrophic spending. Every 1% increase in health spending relative to gross domestic product (GDP) was found to be associated with a reduction of 0.13% in the number of people that incurred catastrophic health spending.

**Conclusion:**

There is substantial variation in financial protection, as measured by the number of people that incurred catastrophic health spending, in LMICs; a proportion of this could be explained by the difference in GDP and external health expenditure.

## Introduction

Attaining Universal Health Coverage (UHC) is a health system outcome, which means access to needed health services by everyone and financial protection from the cost associated with the services received.^1^ This health system goal has been in existence since the 20th century, its origin is linked to the German and the UK health systems when it is commonly referred to as universal health care.^2^^,^^3^ Many countries, especially in Europe, have adapted different aspects of these models to design their health systems and achieved commendable feats by increasing access to health services for their population.^4–6^ Nevertheless, a recent report showed that about 50% of the world’s population still lack access to essential health services and more than one hundred million people slipped into poverty due to health expenditure.^1^ More than 50% of this prevalence was attributed to low- and middle-income countries (LMICs) in Asia, Latin America & Caribbean and Africa.

In 2005, the World Health Organisation (WHO) mobilized all its member states to commit to advancing the concept in their respective countries under a new term called UHC.^2^^,^^7^ It has become one of the pillars of global health and development goals in recent years; the two components of this concept are now part of the sustainable development goals (SDG), with a target of 80% health service coverage and 100% financial protection by 2030.^8^^,^^9^ However, there have been some disagreements over this concept, and its definition being considered ambiguous and broad by some experts. Some argued that in countries with limited resources and weak health system, it is impossible or unsustainable to provide universal access to health services. Experts with this opinion are called the selective or vertical ideologist in global health, they claim that realistic strategies to achieving outcomes such as UHC is by focusing on selected and evidence-base priorities. Except for the current SDGs, this ideology dominates most of global health priorities from 1978 Alma-Ata declaration of PHC to the Millennium Development Goals (MDGs).^6^^,^^10^ However, being selective defeats the UHC goal in itself. On the other hand, the horizontal or system ideologist strongly advocate for comprehensive approaches by strengthening health systems and projecting health as basic human right. This ideology aligns more with UHC goals but there are many challenges ranging from sustainability, vertical forms of development assistance to funding from donor agencies that is prominent in many LMICS.^10^^,^^11^

WHO and the World Bank as the main global institutions championing UHC implementation in LMICs have argued that the most effective pathway to achieving UHC is by significant reduction in out-of-pocket (OOP) health expenditure through introduction of prepayment schemes.^12^^,^^13^ LMICs such as Rwanda, Mexico, Ghana, Brazil and Malaysia have taken great strides towards achieving the health service coverage target; some of which have received international commendation and projected as trailblazers for the UHC concept.^14^^,^^15^ However, access to health service does not translate to protection from financial risk associated with the use of health services^16^; therefore, the overarching goal of the UHC agenda might be truncated if the financial protection component is ignored or not given adequate attention in research and implementation.

Globally, the percentage of health spending by individuals at the point of receiving health services, called OOP payment, has declined. However, the out of pocket health expenditures as a percentage of individuals’ income or consumption is not reducing.^17^ Also, reports have shown that OOP payment for health services contributes to increased financial hardship and global poverty rate significantly although this varies between countries with the highest statistics found LMICs. Therefore, financial protection as a key to achieving UHC has received increased attention, especially in the LMICs.

Financial protection is assessed and measured by two indicators, namely; catastrophic health spending (defined as household health expenditure exceeding 10 or 25% of consumption or income) and impoverishing health spending (defined as expenditure on health care that pushes households below the poverty line).^1^ Meanwhile, investigating the variation of health indicators and outcomes has been found useful in epidemiological studies, especially in health inequalities and health system research. The Shewhart and Deming’s approach to examining variation has gained increased attention recently. According to this approach, variations are the result of multiple factors subjected to common and special-cause.^18^^,^^19^ Therefore, common-cause variation is conceptualized as expected causes that are part of every process or system while special-cause variation is unusual and they are credited to special conditions outside the process and system. As most LMICs are faced with similar health system, economic and development challenges,^20^^,^^21^ we used the approach above to explore the variation in financial protection across selected LMICs; we also examined the underlying country level-factor that might explain the variations.

## Methods

This ecological study uses the most recent datasets available between 2000 and 2016 in the Global Health Expenditure Database (WHO 2014) and the World Development Indicators.^17^ Data from the former is based on relevant national representative cross-sectional surveys such as household income and expenditure surveys, house living standard and socioeconomic surveys, while data from the later database are majorly derived from administrative and validated data from government agencies. Estimates from models based on trends and historical data are sometimes used in both when data are unavailable. Detailed descriptions of these data collection procedures are described elsewhere.^8^^,^^22^

### Study variables

Dependent variables: Catastrophic health spending, in accordance to the SDG framework was used as the indicator to measure financial protection. Therefore, catastrophic health spending defined as the numbers of people that spent more than 10% of their income or household consumption on OOP health care was used. The actual numbers of people that incurred catastrophic spending was preferred to their percentage as more countries have this indicator and it is more suitable for specific analysis performed.

**Table 1:** Descriptive table of dependent and independent variables

*Variables*	*Mean*	*SD*	*Min*	*Max*
Number of people that incurred Catastrophic health spending (Billion)	0.12	45.68	454.49	25.94
Adult population (millions)	46.98	146.89	74467	991.99
GDP (Billion)	0.38	1.9	4045	12.40
HDI rank	0.62	0.13	0.31	0.81
HOOP (Million)	7.8	40.25	8.7	301.26
CHE (as % of GDP)	5.67	2.22	2.31	13.28
OOPS (as % CHE)	41.15	19.05	6.37	87.10
ExT (as % of CHE)	10.94	14.26	0	58.65
SHI (as % CHE)	13.20	19.50	0	69.48
VHI(as % CHE)	3.54	5.40	0	35.11

Independent variables: The following country-level variables were used to examine the underlining factor for variation in financial protection: Adult Population in Million, Gross Domestic Product (GDP) and Human Development Index (HDI). Others are health financing variables described below:

- ▪ Current Health Expenditure as % Gross Domestic Product (CHE%GDP): This indicator is used to indicate a country’s spending on health relative to its economic size.
- ▪ External Health Expenditure from External Sources as % of Current Health Expenditure (EXT%CHE): This indicator reflects the dependency of a country’s health system on external sources of funding.
- ▪ Social Health Insurance as % of Current Health Expenditure (SHI%CHE): This indicates the share of current health expenditure contributed by prepaid contribution to compulsory insurance schemes in each country
- ▪ Voluntary Health Insurance Contributions as % Current Health Expenditure (VHI%CHE): This indicates the share of current health expenditure (CHE) attributable to prepaid private contribution to voluntary insurance schemes
- ▪ Household out-of-pocket payment (HOOP): It is the direct payment made at the point of purchase for healthcare services from household consumption.
- ▪ Out-of-Pocket Expenditure as % Current Health Expenditure (OOP%CHE): This measure the percentage of overall CHE contributed by OOP payment at the point of receiving care and services.

The list of selected LMICs, year of data used, number of people that incurred catastrophic spending and current health expenditure as % of GDP were presented in the Supplementary material (Appendix I).

### Statistical analysis

Summary statistics were used to show the distribution of the main variables, the values were expressed as absolute number with percentages and mean with standard deviation (SD) for categorical and continuous variables, respectively. We generated scatter plots of catastrophic cost, as a percentage, against the number of the adult population (the denominator for the percentage). The mean state performance and exact binomial three sigma limits were calculated for all possible values for the number of cases and used to create a funnel plot. We used Pearson’s correlation analysis to examine the association between the dependent and independent variables. Factors associated with variation in catastrophic expenditure were explored using univariable and multivariable regression models for count outcomes. Univariable negative binomial regression analyses were used to investigate the unadjusted (crude) associations and multivariable negative binomial regression analyses were used to determine variables independently associated with the variation in catastrophic expenditure. The adult population included as the offset variable in our models. Variables with *P*-values less than 0.05 in the univariable analyses were included in the multivariable model. All tests were two-sided and statistical significance was defined at the 5% alpha level. Scatter plots with prediction line were further used to check the liner regression assumptions for the variables present in the final model. Finally, multicollinearity test was conducted using the variance inflation factor, the mean values of all independent variables were significant as the mean values were less than 2, showing insignificant multicollinearity. Data were processed and analyzed with R 3.61 version.

### Ethical approval

This study was based on secondary datasets which were all publicly available and anonymous; therefore, ethical approval is not required for this study.

## Result

### Descriptive statistics

**Fig. 1:**
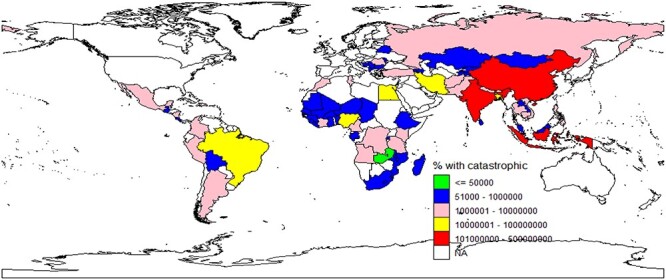
Map showing the variation in number of people that incurred catastrophic spending in LMICs.

The summary statistics (Table 1) includes the mean, SD and range of all the variables based on data from 75 LMICs. The average adult population from the 75 LMICs is about 46 million, ranging from about 75 000 in Sao Tome Principle to about 1Billion in China; on the average, 12 million people incurred catastrophic health spending ranging from 1000 to 256 Million, Figure 1 showed a graphical representation of the variation. The average GDP across the LMICS is about 0.38 Billion, and average HDI is 0.62. On the average, only about 5.67% of GDP is attributable to CHE, HOOP payment (OOPS%CHE) contributed the highest percentage (41.15%) followed by social health insurance and external health expenditure (13.20 and 10.94%, respectively) while voluntary health insurance schemes contributes the lowest (3.54%). The average HOOP across the 75 LMICs is about 8.5 million ranging from 9 to about 300 million NCU.

**Fig. 2:**
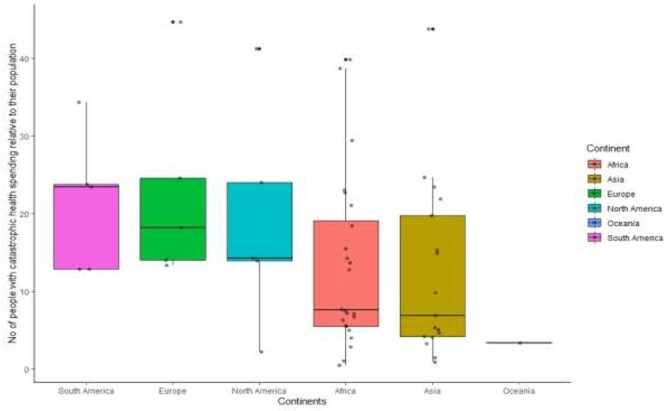
Boxplot showing the variation between continents and the no of people with catastrophic spending on health.

Further descriptive analysis using boxplot (Fig. 2) to explore the variation in financial protection among the selected countries revealed that the number of people that experienced catastrophic health spending relative to individual their adult population is highest in South America, followed by Europe, and the least in Oceania. Figure 1 further shows the variation in number of people that incurred catastrophic health spending across the selected 75 LMICs.

#### Special-cause and common-cause variation in financial protection

**Fig. 3:**
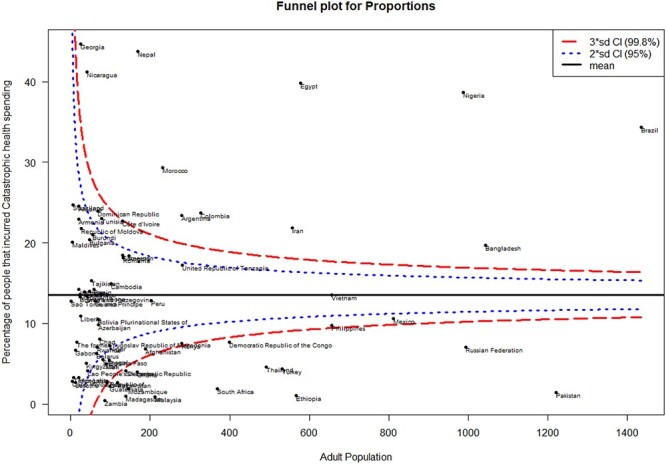
Funnel plot showing common and specific variation in catastrophic spending in LMICs.

Figure 3 shows the result of the funnel plot which explored variation in financial protection in 75 LMICs. An average of 8% of the entire population incurred catastrophic health spending. A visual inspection of the funnel plots identifies 38 (46%) countries within the 99% control limits indicating common-cause variation. Eleven (13.9%) countries were above the upper control limit (higher than the average) and 15 (19%) countries were below the lower control limit (lower than the average), both indicating special-cause variation in the number of people that incurs catastrophic health spending.

#### Association between financial protection and country-level factor

The result from the regression analyses to examine the variation in financial protection across LMICs and associated country-level characteristics is shown in Table 2. After controlling for the confounding effect of other variables in the multivariable (adjusted) regression model, only four out of the eight variables examined (GDP, CHE%GDP, ExT%CHE and HOOP) were found to have independent and significant associations with variation in catastrophic spending (Table 2). The result shows that CHE%GDP—which is country’s spending on health relative to its economic seize—was the strongest independent underlying factor for variation in catastrophic health spending among the included LMICs. Every 1% increase in CHE%GDP decreases the number of people that spent over 10% of their income or consumption on health expenditure by 0.13%. Furthermore, every 1% increase in GDP and external funding towards health (ExT) also decreases catastrophic spending incurred by household by 0.06 and 0.09%, respectively (Table 2). In our model, only HOOP was found to have a positive effect on number of people that incurred catastrophic spending.

**Table 2:** Unadjusted and adjusted regression analysis with independent variables

*Independent variable*	*Unadjusted coefficient and CI at 95%*	*Adjusted coefficient and CI at 95%*
GDP	0.344 (0.189, 0.500)^***^	−0.063 (−0.121, −0.005)^***^
HDI	2.231 (−1.667, 6.129)	—
CHE%GDP	−2.167 (−4.140, −0.194)^**^	−0.130 (−0.229, −0.032)^**^
OOPS	0.861 (−0.830, 2.552)	—
ExT	−0.336 (−0.634, −0.038)^**^	−0.013 (−0.026, 0.001)^*^
SHI	0.197 (−0.070, 0.463)	—
VHI	0.283 (−0.239, 0.805)	—
HOOP	0.334 (0.179, 0.488)^***^	0.085 (0.029, 0.141)^***^

^*^0.1, ^**^0.05, ^***^0.01

## Discussion

### Main findings of this study

This ecological study was conducted to explore the variation in financial protection in LMICs and examine country-level characteristics associated with observed variations. This study revealed that there is a wide variation in financial protection across the 75 included LMICs; 55 countries exhibited common-cause variation. Some countries such as China and India have as high as 200million people spending more than 10% of their income or consumption on health expenditure, while countries such as Djibouti has as small as 9000 that incurred catastrophic health spending. In the regression analysis, health spending by government relative to their economic seize (CHE%GDP) was the strongest underpinning factors for variation in financial protection in LMICs. Along with ExT%GDP and GDP, an increase in CHE%GDP reduces catastrophic health spending. On the other hand, HOOP contributed to the variations positively, it also suggested that an increase in HOOP expenditure on health increases catastrophic health spending.

### What is already known on this topic

These findings are consistent with other global and regional studies that have examined and reported a similar pattern of variation in the prevalence of financial protection.^1^^,^^7^^,^^16^^,^^23^ Most LMICs are faced with similar health and economic challenges,^21^ therefore, it is expected that more people will incur catastrophic health spending in countries with a larger population as observed in this study. Therefore, focusing on the number of people that incurred catastrophic spending alone might be misleading and thus lead to inaccurate conclusions. The map in Fig. 1 and box plot in Fig. 2 clearly reflected this, people incurred catastrophic spending in the LMICs in South America and in some European countries when adjusted by their adult population; the LMICs in Oceania had the lowest.

A recent study^16^ reported that a higher proportion of people from Asia and Africa incurred catastrophic health spending and slipped into poverty (at $1.90) due to health expenditure. These findings are not consistent with our descriptive result when we adjusted for adult population in box plot and when proportion of population that incurred catastrophic spending was used in Figure 1. Unlike our study, the paper focused on all countries irrespective of their income classification. In addition, they used health spending more than “10% of total consumption” to measure catastrophic health spending while our study was based on “health spending more than 10% of income or household consumption”. The differences in methods and population of interest might have contributed to the contrast in our findings, nevertheless, it is worth mentioning that the paper also reported an increase in the proportion of the population that incurred catastrophic spending in Europe and Latin America. In addition, the recent global monitoring report on UHC reported Latin America to have the highest rate of OOP expenditure exceeding 10%.^1^

Both common-cause and specific-cause variations were observed in the funnel plot analysis, variations in financial protection in most of the LMICs were attributable to common-cause factors. However, countries such as Nigeria, Brazil, Egypt, Nepal and Georgia with a very high prevalence of catastrophic spending (above 30%) all showed special-cause variability. A more rigorous epidemiological study design will be suitable to elicit specific factors associated with a higher prevalence of catastrophic health spending and possibly compare with countries with very low prevalence as this will further inform and strengthen policy design and implementation.

In the multivariate regression analyses, GDP, which indicates the economic strength of each country, was one of the indicators of interest. We found that it was independently associated with variation in the number of people that incurred catastrophic health expenditure among the 75 LMICs (Table 2). Wagstaff *et al.*^16^ also reported similar findings in their study—which examined the incidence of catastrophic spending in 133 countries, irrespective of their economic strength. Other previous studies have also indicated that catastrophic spending decreases as the percentage of government spending relative to GDP increases.^15^^,^^24^ We found that health expenditure at country level relative to their economic seize (CHE%GDP) is the strongest underpinning country-level factor associated variation in the number of people that incurred catastrophic health spending. A recent study on domestic health spending of 195 countries by the Global Burden of Disease Health Financing Collaborator Network showed a steady increase global health financing over the last two decades.^25^ Although, the pace of growth is slow and disparities in per-capita health spending persist, growth is projected to continue, probably doubles and with more impact in countries with lowest health expenditure now. In LMICs, health sectors continue to grow faster than most economy, for example, Abuja 2001 Declaration captures how African head of states pledged minimum of 15% of annual budget for health spending.^26^ Even though just few countries have met with this goal, a recent study by the WHO show that the minimum percentage of health spending in LMICs increases by 6% per year.^22^ Attaining UHC involves several political and inter-agency processes that are capable of facilitating or inhibiting its achievement, and this is largely determined key stakeholders engagement and acceptance. Therefore, there is need for heavy investment on policymakers and key stakeholders to develop their political will and capacity for implementation of health expenditure policies to support UHC agenda in LMICs. One of such platforms is the Joint Learning Network for Universal Health Coverage (JLN); the network is actively involved in training and development of policymakers and practitioners, with help from global partners, to facilitate development of innovative techniques in advancing UHC in LMICs.

Many LMICs including those involved in this study have embarked on increasing government health spending through the implementation of national health insurance schemes.^27–29^ However, the sustainability of these schemes has been subject of discussion in recent years especially in LMICs with limited or scares resources.^30^^,^^31^ A common component of these schemes across countries is the compulsory donation by citizens in the form of tax or monthly premium payment to the government—which is usually voluntary. Therefore, a significant reduction in the number of people that incurs catastrophic health spending is expected in a healthcare system with health insurance schemes.^32^^,^^33^ Studies on equity of insurance schemes have reported that premium payments are usually progressive than insurance scheme funding based on tax—which is usually compulsory.^34^^,^^35^ Findings of this study, we found that the share of CHE contributed by compulsory (SHI%CHE) and voluntary (VHI%CHE) payment into health insurance scheme was not significantly associated with the variation in the number of people that incurred catastrophic health spending. This also suggests that both compulsory and voluntary health insurance schemes are similar in most LMICs. The weak governance structures and corruption have been identified as a constant bane affecting healthcare system of most LMICs.^36^^,^^37^ Corruption in particular is fuelled by presence of many actors—suppliers, regulators, provides and consumers—who had to interact at different levels. Before the emphasis on UHC became global, report from few studies have shown how developing countries in Latin America, Asian and Africa subtly incorporate principles of marketing in health services, treating health as a commodity.^38^^,^^39^

HOOP was also found as one of the significant indicators associated with variation in number of people that incurs catastrophic health spending in LMICs. In consistence with other global studies,^1^^,^^15^ the result showed that every 1% increase in HOOP within the LMICs increased the number of people that incurred catastrophic spending by 0.085%. Global estimate as reported in the 2010 World Health report revealed that decline in catastrophic health spending can only be significant when the percentage of total health expenditure contributed by HOOP falls below 15–20%.^40^ In this study, the average OOP payment as a percentage of current health expenditure (OOP%CHE) was considerably high (40%); a high percentage of OOP payment have also been reported among LMICs in previous studies.^16^^,^^23^ This might suggest why OOPS%CHE was not significantly associated with the variation in the number of people that incurred catastrophic health spending among the LMICs included in this study because their prevalences are similar.

Finally, we also discovered that the degree of dependency on external funding through foreign governments and donor agencies is independently associated with variation in catastrophic spending. The more an LMIC depend on external funding, the lesser the number of people that incurred catastrophic spending in such country. This finding strongly supports the narrative that external funding or donor agencies significantly contributes to increased access to basic health care services in many LMICs.^41^ OOP spending will remain substantial in LMICs as many are dependent on development assistance although with more health spending by the government, huge investment in health is feasible. This is usually made possible by providing reduced and sometimes free but effective health services in areas such as antenatal care, and free immunization, vaccination and screening health services to a large population. Globally, development assistance for health (DAH) has been reported to have increased dramatically especially since the Millennium Development Goal era.^42^^,^^43^ However, concerns have been raised about the poor alignment of priorities between funding agencies and recipient countries, especially in a limited-resource setting.^43^^,^^44^ The question is, what happens at the end of grant year or project activities by the donor agencies? Increasing the efficiency of current spending on health and development assistance is key to sustainable health development especially in the absence of new and sustained health funding.^25^ In addition, evidence-based advocacy aimed at convincing relevant governmental stakeholder in health system to recognize that healthier citizens contribute massively to development is important. This coupled with the synchronization of funder/donor agencies programs with countries development agenda might guarantee more domestic commitment, investment and sustainability of health interventions.

### What this study adds

This study showed that variation in financial protection in LMICs is huge, with more than two-thirds (67%) of the included LMICs exhibiting common-cause variations. This again suggests that most LMICs are faced with similar health system challenges in combating financial risk when assessing health services. Further analysis showed that a considerable part of this variation is accounted for by difference in CHE%GDP, CHE, HOOP and ExT. We strongly suggest that interventions aimed at improving socioeconomic development and healthcare funding and implementation of effective health insurance schemes targeted at the disadvantaged are keys to mitigating the number of people that incurred catastrophic health expenditure in LMICs. There is need for purposeful effort to make LMICs, especially those in Africa and South Asia, less dependent on aid and external donors. Finally, we suggest that rigorous epidemiological techniques should be employed to identify and unpack the variation in catastrophic health spending in countries that exhibited special-cause variations in our study.

### Limitation of this study

The datasets used for this study were from reliable sources as the raw data used are from national representative surveys and rigorous statistical techniques were applied when necessary. However, the findings from this study are exposed to ecological inference fallacy; therefore, the findings from the regression analyses do not represent causative relationships but associations. In addition, the data used are from two different databases, data collection procedure might be different. However, effort was made to minimize heterogeneity in our data by extracting and merging same year data point for each country involved in our analysis.

## Supplementary Material

Supplementary_fdab021

## Notes

**Seun S. Anjorin,** Mr.

**Abimbola A. Ayorinde,** Dr.

**Mustapha S. Abba,** Mr.

**Oyinlola O. Oyebode,** Dr.
