# Corrigendum to: Variation in financial protection and its association with health expenditure indicators: an analysis of low- and middle-income countries

**DOI:** 10.1093/pubmed/fdab310

**Published:** 2021-07-15

**Authors:** Seun S Anjorin, Abimbola A Ayorinde, Mustapha S Abba, Oyinlola O Oyebode, Olalekan A Uthman

**Affiliations:** Population Evidence and Technologies, Division of Health Sciences, Warwick Medical School, University of Warwick, Coventry CV4 7AL, UK; Warwick-Centre for Applied Health Research and Delivery (WCAHRD), Warwick Medical School, University of Warwick, Coventry CV4 7AL, UK; Population Evidence and Technologies, Division of Health Sciences, Warwick Medical School, University of Warwick, Coventry CV4 7AL, UK; Warwick-Centre for Applied Health Research and Delivery (WCAHRD), Warwick Medical School, University of Warwick, Coventry CV4 7AL, UK; Warwick-Centre for Applied Health Research and Delivery (WCAHRD), Warwick Medical School, University of Warwick, Coventry CV4 7AL, UK

In the originally published version of this manuscript, there was an error in the title. The title should read: ``Variation in financial protection and its association with health expenditure indicators: an analysis of low- and middle-income countries'', instead of: ``Variation in financial protection and it association with health expenditure indicators: an analysis of low- and middle-income countries''. This error has been corrected online and in print.

