# Supplementary material for: Variation in financial protection and its association with health expenditure indicators: an analysis of low- and middle-income countries
Source: J Public Health (Oxf). 2021 Apr 23;44(2):428–37. doi: 10.1093/pubmed/fdab021 (PMC9234505; doi:10.1093/pubmed/fdab021)
Supplement: Supplementary_fdab021 [file supplementary_fdab021.docx]

Appendix – List of selected LMICs, Year of data used, no of people that incurred catastrophic spending and current health expenditure as % of GDP

| S/N | *Country* | Year | No of people that incurred Catastrophic health spending | CHE_GDP |
| --- | --- | --- | --- | --- |
|  | *Afghanistan* | 2013 | 1288000 | 8.80594063 |
|  | *Angola* | 2008 | 2694000 | 3.32290483 |
|  | *Armenia* | 2013 | 464000 | 10.32344532 |
|  | *Azerbaijan* | 2005 | 682000 | 7.43525314 |
|  | *Bangladesh* | 2016 | 37435224 | 2.31177998 |
|  | *Belarus* | 2016 | 414000 | 5.91054153 |
|  | *Benin* | 2011 | 835000 | 4.22198343 |
|  | *Bolivia Plurinational States of* | 2015 | 520905 | 6.61701393 |
|  | *Bosnia and Herzegovina* | 2015 | 30160 | 9.33953667 |
|  | *Brazil* | 2008 | 49300000 | 8.01595783 |
|  | *Bulgaria* | 2010 | 950000 | 7.10581446 |
|  | *Burkina Faso* | 2014 | 473946 | 5.62491608 |
|  | *Cabo Verde Republic of* | 2007 | 10000 | 4.18900156 |
|  | *Cambodia* | 2014 | 2151164 | 6.70157766 |
|  | *Cameroon* | 2014 | 2398000 | 4.53427935 |
|  | *Chad* | 2003 | 587000 | 5.50941277 |
|  | *China* | 2013 | 259444382 | 4.71002245 |
|  | *Colombia* | 2016 | 3761010 | 7.21367979 |
|  | *Costa Rica* | 2012 | 454.487 | 7.91350985 |
|  | *Côte d'Ivoire* | 2015 | 2421859 | 4.42198181 |
|  | *Democratic Republic of the Congo* | 2012 | 2554351 | 3.56752944 |
|  | *Dominican Republic* | 2007 | 1678687 | 4.20731974 |
|  | *Egypt* | 2012 | 2.3e+07 | 4.55305433 |
|  | *Ethiopia* | 2015 | 3658548 | 3.9449203 |
|  | *Gabon* | 2005 | 80000 | 2.93410254 |
|  | *Georgia* | 2013 | 1103000 | 8.3957243 |
|  | *Ghana* | 2012 | 239488 | 4.11767912 |
|  | *Guatemala* | 2014 | 216000 | 5.94237423 |
|  | *Guinea* | 2012 | 786000 | 3.50398302 |
|  | *India* | 2011 | 2.16e+08 | 3.24634194 |
|  | *Indonesia* | 2016 | 2.16e+08 | 3.11905479 |
|  | *Iran* | 2013 | 12200000 | 5.99379873 |
|  | *Jamaica* | 2004 | 278000 | 5.25798988 |
|  | *Kazakhstan* | 2015 | 436459 | 3.0457468 |
|  | *Kenya* | 2015 | 1956849 | 5.21938562 |
|  | *Kyrgyzstan* | 2016 | 191144 | 6.41248226 |
|  | *Lao People's Democratic Republic* | 2007 | 177000 | 3.15345573 |
|  | *Lesotho* | 2010 | 85664 | 7.61773348 |
|  | *Madagascar* | 2010 | 300311 | 5.32034874 |
|  | *Malawi* | 2016 | 639201 | 9.69363403 |
|  | *Malaysia* | 2004 | 186000 | 2.91764545 |
|  | *Maldives* | 2009 | 71016 | 10.0928812 |
|  | *Mali* | 2016 | 856267 | 3.77538157 |
|  | *Mauritania* | 2014 | 355142 | 4.28468752 |
|  | *Mexico* | 2016 | 1872391 | 5.61609173 |
|  | *Mongolia* | 2013 | 67000 | 4.06732368 |
|  | *Morocco* | 2006 | 6792000 | 4.94123936 |
|  | *Mozambique* | 2014 | 369352 | 5.9676466 |
|  | *Nepal* | 2014 | 2894161 | 5.76784754 |
|  | *Nicaragua* | 2014 | 884427 | 8.0098114 |
|  | *Niger* | 2011 | 1128917 | 6.6486249 |
|  | *Nigeria* | 2012 | 23219305 | 3.35984278 |
|  | *Pakistan* | 2015 | 7594990 | 2.68711925 |
|  | *Peru* | 2016 | 2653317 | 5.0635891 |
|  | *Philippines* | 2015 | 6419000 | 4.32136869 |
|  | *Republic of Moldova* | 2016 | 664980 | 7.53593349 |
|  | *Romania* | 2016 | 2647706 | 4.99375629 |
|  | *Russian Federation* | 2014 | 7005000 | 5.18879557 |
|  | *Rwanda* | 2016 | 117744 | 6.76334143 |
|  | *Sao Tome and Principe* | 2000 | 14000 | 10.50688744 |
|  | *Senegal* | 2012 | 443000 | 4.22773361 |
|  | *Serbia* | 2015 | 583519 | 8.81929111 |
|  | *Sierra Leone* | 2011 | 2822981 | 13.28134346 |
|  | *South Africa* | 2010 | 717000 | 7.41535139 |
|  | *Sri Lanka* | 2016 | 1085881 | 3.89324164 |
|  | *Swaziland* | 2009 | 158000 | 8.63292217 |
|  | *Tajikistan* | 2009 | 1267773 | 5.85579777 |
|  | *Thailand* | 2009 | 99614 | 3.61940002 |
|  | *The former Yugoslav Republic of Macedonia* | 2008 | 112000 | 6.55317593 |
|  | *Timor-Leste* | 2014 | 23000 | 2.36415315 |
|  | *Tunisia* | 2015 | 192801 | 7.00741243 |
|  | *Turkey* | 2016 | 2312000 | 4.31431437 |
|  | *United Republic of Tanzania* | 2011 | 186120 | 5.12877369 |
|  | *Vietnam* | 2016 | 8521831 | 5.65919352 |
|  | *Zambia* | 2010 | 40000 | 3.71929598 |
